# Early and Mid-term Outcomes in Female Patients Undergoing Isolated Conventional Coronary Surgery

**DOI:** 10.5681/jcvtr.2014.023

**Published:** 2014-06-30

**Authors:** Kazim Erguneş, Levent Yilik, Ufuk Yetkin, Banu Lafcı, Serdar Bayrak, Berkan Ozpak, Ali Gurbuz

**Affiliations:** Izmir Katip Celebi University Ataturk Training and Research Hospital, Department of Cardiovascular Surgery , Izmir, Turkey

**Keywords:** Female Gender, Coronary Surgery, Predictive Factors, Postoperative Period, Survival

## Abstract

***Introduction:*** Several observational studies comparing outcomes between female and male patients after coronary artery bypass grafting (CABG) have shown that operative mortality rate is higher among female patients than in male patients. However, some conflicting studies report that early mortality among female patients is equivalent to that among male patients. We investigated predictive factors of morbidity, mortality and survival in female patients undergoing isolated conventional CABG.

***Methods:*** Between January 2002 and December 2009, 1657 patients underwent isolated conventional CABG in our clinic. 21.8% (n=361) of patients were female and 78.2% (n=1296) males.

***Results:*** Advanced age (P<0.0001), hypertension (P<0.0001), diabetes (P<0.0001), and hyperlipidemia (P<0.0001) were the independent predictive factors among female patients. Mean in-hospital mortality rates were 5.8% and 3.2%; for females and males, respectively (P=0.029). Prolonged ventilatory support (P=0.009) and postoperative atrial fibrillation (P=0.049) were the independent predictive factors of in-hospital mortality in female patients. Cardiopulmonary bypass time (P=0.041), prolonged ventilatory support (P<0.0001), and postoperative atrial fibrillation (P=0.031) were the independent predictive factors of in-hospital mortality in male patients. Mean follow-up was 47.51±25.06 months and 48.42±25.21 months among female and male patients (P=0.820). In follow-up, mortality rate was 6.1% (n=22) among female patients and 4.6% (n=60) among male patients (P=0.272). Left internal thoracic artery (LITA) usage (P=0.001) was the independent predictive factor of survival in female patients.

***Conclusion:*** In-hospital mortality rate was higher in female patients. Length of ICU and hospital stay, and mid-term survival was similar between female and male patients.

## Introduction

Despite the decrease in operative mortality and morbidity rate in patients undergoing isolated on-pump coronary artery bypass grafting (CABG) in recent years, several observational studies comparing outcomes between female and male patients after CABG have shown that operative mortality rate is higher among female patients than in male patients.^1-4^ However, some conflicting studies report that early mortality among female patients is equivalent to that among male patients.^5,6^

Conflicting results among published studies with regard to the influence of gender on postoperative morbidity and mortality after CABG are influenced by a number of factors: dissimilar study designs and data collection method, different study sample sizes, and variable application of statistical methods to analyze the data sets.

In our study, we investigated predictive factors of morbidity, mortality and survival in female patients undergoing isolated conventional CABG.

## Materials and methods

Between January 2002 and December 2009, 1657 patients underwent isolated CABG in our clinic. 21.8% (n=361) of patients were female and 78.2% (n=1296) male.

Cases of redo-CABG surgery, off-pump CABG surgery, simultaneous cardiac valve surgery, simultaneous carotid endarterectomy, simultaneous ventricular aneurysm surgery and simultaneous peripheral vascular surgery were not included in the study.

### Perioperative management

Anesthetic, surgical, and CPB techniques were similar for all patients. The method of anesthesia was equally applied to all cases. Induction of anesthesia was provided through titration of 2 mg of midazolam I.V, 2-5 μg/kg of fentanyl I.V and 2-5 mg/kg of thiopental sodium I.V. Muscle relaxation was achieved by 0.1 mg/kg pancuronium bromide and anesthesia was maintained by high dose fentanyl. Cardiopulmonary bypass was undertaken using a standardized extracorporeal circulation utilizing non-pulsatile flow, and an alpha-stat management protocol.

The systemic perfusion was performed using hypothermic bypass strategy of systemic cooling between 28 ^°^C and 32 ^°^C. Isothermic blood cardioplegia was initially administered antegradely and retrogradely, and thereafter continuously retrogradely. After the surgery, patients were transported to the intensive care unit and followed up according to the unit protocol. Prolonged CPB time was defined as CPB time >3 hours. Prolonged inotropic drug support was defined as use of inotropic drug for >24 hours.

Prolonged ventilatory support was defined as pulmonary insufficiency requiring ventilatory support >24 hours postoperatively. Prolonged ICU stay was defined as ICU stay of >48 hours, while normal ICU stay was defined as ICU stay lasting ≤48 hours. Prolonged hospital stay was defined as hospital stay of >5 days. Preoperative, operative, and postoperative data of the patients were collected prospectively and entered into our cardiac surgery registry. Data of death and cause of death were obtained through hospital records and physician records. Information was obtained through phone calls with patient families, or the patient’s family physician or cardiologist.

### Statistical Analysis

Following data accumulation, statistical models were formed and analysed using SPSS 15 (SPSS Inc, Chicago, The United States). Non-categorical data were analysed with t-test and categorical data with Chi-Square or Fisher’s Exact test where appropriate. Mann-Whitney was used in analysing duration of intensive care unit stay and hospitalization periods. Survival analysis was performed using the Kaplan-Meier method. Log-Rank test was used in analysis of risk factors affecting survival. Variables with a P value equal or less than 0.05 were entered into the multivariate regression model. Independent predictors of duration of ICU and hospital stay were analyzed using a multivariate linear regression analysis. Independent predictors of postoperative mortality were analyzed using a multivariate logistic regression analysis. In multivariate analysis, independent predictors of survival were analyzed using Cox-regression analysis.

## Results

Advanced age, hypertension, diabetes, and hyperlipidemia were more common among female patients. Smoking and peripheral vascular disease were more common among male patients (Table 1 ).

**Table 1:** Preoperative data for female and male patients

	**Male**	**Female**	**P value**
	**(N=1296)**	**(N=361)**	
Age (yrs)	57.71±10.74	63.18±9.53	<0.0001
Smoking	69.1 % (896)	16.3% (59)	< 0.0001
Diabetes	32.3% (418)	47.9 % (173)	<0.0001
Hyperlipidemia	42.8 % (555)	53.2 % (192)	<0.0001
Hypertension	40.2% (521)	67.3% (243)	<0.0001
COPD	4.6% (59)	4.2% (15>)	0.885
LMCAD	6 % (78)	5 % (18)	0.525
Renal failure or creatinine ≥2 mg/dl	1.1% (14)	1.9%(7)	0.191
Peripheral vascular disease	5.6 % (73)	0.8% (3)	<0.0001
Preoperative AF	1.3% (17)	1.7 % (6)	0.612
Previous PTCA	5.6% (72)	4.7% (17)	0.599
Previous stroke or TİA	0.7% (9)	1.1% (4)	0.497
Aort calcification	3.1% (40)	3.9 % (14)	0.502
Ejection fraction (%)			0.139
<30	6.6 % (85)	5% (18)	
30-50	40.7% (523)	46.1% (166)	
>50	52.7% (678)	48.9 % (177)	

AF: Atrial fibrillation, COPD: Chronic obstructive pulmonary disease, LMCAD: Left main coronary artery disease, TIA: Transient ischemic attack

Chronic obstructive pulmonary disease (COPD), Left main coronary artery disease (LMCAD), preoperative renal disease, preoperative atrial fibrillation, previous stroke or trancient ischemic attack, previous PTCA, preoperative ejection fraction rates were similar between female and male patients (Table 1).

The mean cardiopulmonary bypass was 98.84 ± 31.82 minutes and 100.81 ± 33.91 minutes among male and female patients, respectively (P=0.304 ). The mean cross-clamp time was 42.23 ± 12.99 minutes and 42.60± 12.77 minutes among male and female patients, respectively (P=0.623). Postoperative blood transfusion (≥3 units) requirement and renal insufficiency were more common among female patients (Table 2).

**Table 2:** Intraoperative, and postoperative data for female and male patients

	**Male**	**Female**	**P**
	**(N=1296) **	**(N=361)**	
Urgent and emergent operations	1.2 % (15)	-	0,052
** CPB time**			0.076
< 3 hour	96.2% (1247)	93.9% (339)	
>3 hour	3.8% (49)	6.1% (22)	
**Number of bypass grafts**			0.892
1	6.3% (82)	6.1% (22)	
2	35.5% (460)	36.8% (133)	
≥3	58.2% (754)	57.1% (206)	
**Cross-clamp time**			0.357
< 1 hour	93.1% (1207)	91.7% (331)	
> 1 hour	6.9% (89)	8.3 % (30)	
**Duration of surgery**			0.037
<3 hour	39.4% (510)	46.3% (167)	
≥ 3-4 hour	51.9% (672)	44.3% (160)	
>4 hour	8.8% (114)	9.4% (34)	
Prolonged intrope use	15.5% (201)	15.5% (56>)	1.000
Intra-aortic balloon support	7.6% (98)	10% (36)	0.155
Prolonged ventilatory support	3.1% (40)	5% (18)	0.104
Postoperative renal failure	2.8% (36)	5.8% (21)	0.008
Postoperative dialysis	1.2% (15)	2.5% (9)	0.078
Postoperatif atrial fibrilasyon	16.3% (211)	16.9% (61)	0.810
Hemorrage-related reexploration	2.5% (32)	2.8% (10)	0.708
**Postoperative blood transfusion **			
Requirement ≥3 unite	4.8% (62)	7.5% (27)	0.048
Postoperative stroke	1% (14)	0.5% (2)	0.503
Mean ICU length of stay (days)	2.67±3.44	2.69.±2.07	0.879
Mean hospital length of stay (days)	6.40±2.16	6.34.±1.40	0.224
In-hospital mortality	3.2% (42)	5.8 % (21)	0.029

AF: Atrial fibrillation, CPB: Cardiopulmonary bypass, ICU: Intensive care unit

The independent predictive risk factors among female patients were shown in Table 3. The independent predictive factors of length of ICU stay among female patients were shown in Table 4. The independent predictive factors of length of hospital stay among female patients were shown in Table 5 . Mean in-hospital mortality rates were 5.8% and 3.2%; for females and males, respectively which was statistically significant (P=0.029).

**Table 3:** Independent predictive factors by multivariete regression analysis of female patients undergoing CABG

**Variable **	**Beta**	**Std. Error**	**OR**	**95% CI for OR**	**P value **
Age	0.038	0.007	1.09	1.02 – 1.05	<0.0001
Diabetes	0.64	0.13	1.91	1.47 – 2.46	<0.0001
Hyperlipidemia	0.68	0.13	1.98	1.53 – 2.56	<0.0001
Hypertension	0.95	0.13	2.60	2.01 – 3.37	<0.0001

**Table 4:** Independent predictive factors of length of ICU stay by multivariete regression analysis in female patients

**Variable**	**Beta**	**Std. Error**	**P value **
COPD	1.33	0.44	0.003
Cross-clamping time	0.88	0.36	0.016
Prolonged inotropic support	1.42	0.39	<0.0001
Prolonged ventilatory support	3.00	0.48	<0.0001
Hemodialysis	2.07	0.72	0.004
Postoperative blood transfusion (≥3 unite)	1.39	0.41	0.001
Postoperative stroke	2.59	1.18	0.029

COPD: Chronic obstructive pulmonary disease

**Table 5:** Independent predictive factors of length of in-hospital stay by multivariete regression analysis in female patients

**Variable**	**Beta**	**Std. Error**	**P value **
Diabetes	0.027	0.10	0.009
Previous stroke	2.25	0.55	<0.0001
Cardiopulmonary bypass time	1.22	0.47	0.010
Cross-clamping time	0.70	0.27	0.010
Prolonged inotropic support	1.41	0.27	<0.0001
Prolonged ventilatory support	2.26	0.58	< 0.0001
Postoperative AF	0.66	0.16	<0.0001
Postoperative renal insufficiency	1.28	0.38	<0.001
Hemodialysis	2.31	0.60	<0.0001
Postoperative blood transfusion (≥3 unite)	1.38	0.33	<0.0001
Postoperative stroke	2.52	0.78	<0.001

AF: Atrial fibrillation

Prolonged ventilatory support (Beta 4.69, S.E 1.79, P=0.009, OR 109.50, 95% CI for OR 3.24 - 3694.45) and postoperative atrial fibrillation (Beta 4.09, S.E 2.07, P=0.049, OR 60.03, 95% CI for OR 1.02 - 3510.10) were the independent predictive factors of in-hospital mortality in female patients.

Cardiopulmonary bypass time (Beta 1.55, S.E 0.76, P=0.041, OR 4.73, 95% CI for OR 1.06 – 20.98), prolonged ventilatory support (Beta 2.95, S.E 0.83, P<0.0001, OR 19.27, 95% CI for OR 3.79 – 98.04), and postoperative atrial fibrillation (Beta 1.87, S.E 0.87, P=0.031, OR 6.49, 95% CI for OR 1.18 – 35.75) were the independent predictive factors of in-hospital mortality in male patients.

Causes of in-hospital death among female patients were arrhythmia (n=2, 0.6%), arrhythmia and pneumonia (n=1, 0.3%), pneumonia (n=1, 0.3%), myocardial infarction (n=2, 0.6%), multiorgan failure (n=14, 3.9%), and renal insufficiency (n=1, 0.3%). Mean follow-up was 47.51±25.06 months and 48.42±25.21 months among female and male patients (P=0.820). LITA usage (B -0.39, S.E 0.11, OR 1.48, 95% CI for OR 1.17 – 1.88, P=0.001) was the independent predictive factor in multivariate cox-regression analysis of survival in female patients.

Calcification of ascending aorta (Beta 0.55, S.E 0.18, OR 1.73, 95% CI for OR 1.21 – 2.48, P=0.002), LITA usage (Beta -0.35, S.E 0.06, OR 0.70, 95% CI for OR 0.61 – 0.80, P<0.0001), operation time (Beta 0.22, S.E 0.06, OR 1.25, 95% CI for OR, 1.10 – 1.43, P=0.001), prolonged inotropic support (Beta 0.85, S.E 0.13, OR 2.35, 95% CI for OR 1.81 – 3.06, P<0.0001), and use of IABP (Beta -0.44, S.E 0.16, OR 0.64, 95% CI for OR 0.46 – 0.89, P=0.009) were the independent predictive factors in multivariate cox-regression analysis of survival in male patients. In follow-up, mortality rate was 6.1% (n=22) among female patients and 4.6% (n=60) among male patients (P=0.272).

Causes of death in follow-up among female patients were myocardial infarction (n=6), multiorgan failure (n=14), multiorgan failure and colon carcinoma (n=1), multiorgan failure and larynx carcinoma (n=1).

Causes of death in follow-up among male patients were myocardial infarction (n=14), multiorgan failure (n=43), multiorgan failure and colon carcinoma (n=1), multiorgan failure and larynx carcinoma (n=1), multiorgan failure and bronchial carcinoma (n=1).

## Discussion

Previous studies suggested that female were more likely to present late for diagnosis, interventional cardiology procedures or surgery than males.^4,7^ Previous studies showed that the rate of female gender in patients undergoing CABG surgery was between 19.3% and 30%.^4,8-10^ In our study, 21.8% of patients were female.

Advanced age, hypertension, diabetes, and hyperlipidemia are recognized risk factors for early and late adverse outcome in patients undergoing CABG. It was stated in the literature that female patients undergoing CABG were older,^4,9,12,13^ and had a higher prevalence of diabetes,^4,6,14,15^ hypertension,^10,12,13^ and hyperlipidemia.^9,12^

We stated that advanced age, hypertension, and diabetes were more common among female patients. In our study, the preoperative lipid profiles for female patients were distinctly different from male patients. Hyperlipidemia rate was higher among female patients. We also stated that these risk factors were independent predictive factors among female patients. Previous studies showed that female patients undergoing CABG had higher prevalence of peripheral vascular disease,^9,12,15^ low ejection fraction^12,15^ and had longer CPB time,^4^ cross-clamping time^4^ and had higher inotropic support,^4,5^ and had longer length of hospital stay,^4,9^ but these factors could not be confirmed in our study. Several studies showed that compared with male patients, female patients undergoing coronary surgery were less likely to receive vessel grafts.^4,16^

Some studies showed that rate of use vessel grafts was similar between female and male patients.^13,17^

We found that rate of use of vessel grafts was similar between female and male patients. Several studies showed that risk of morbidity and mortality after coronary artery bypass grafting was higher for women than for men.^1-3,9,18^

Several studies showed that in-hospital mortality rate was between 3.6% and 5.6% among female patients and between 1.4% and 2.9% among male patients.^11,13,19,20^

Some studies showed that incidence of in-hospital mortality was not statistically different between women and men.^6,9^ Our investigation of 1657 patients undergoing coronary surgery found that female patients was so distinctly different from male patients in their preoperative and postoperative profiles.

We stated that in-hospital mortality rate was 5.8% and 3.2% in female and male patients and these findings were significant statistically. Risk factors affecting in-hospital mortality in female patients in a study that could not be confirmed in our study were advanced age, peripheral vascular disease, postoperative blood transfusion, abnormal left ventricular function.^5^

Reasons for increased in-hospital mortality in female patients compared to male patients remain unclear. Conflicting results among published studies with regard to the influence of female gender on postoperative morbidity and mortality after coronary surgery are influenced by a number of factors: associated comorbidities, dissimilar study designs, different study sample sizes, and variable application of statistical methods to analyze the data sets.

We stated that prolonged ventilatory support and postoperative atrial fibrillation were the independent predictive factors of in-hospital mortality in both male and female patients. We also stated that cardiopulmonary bypass time was the independent predictive factors of in-hospital mortality in male patients.

Several studies showed that length of hospital stay was longer among female patients undergoing coronary surgery.^4,9^ We stated that length of ICU and hospital stay was not significantly different between female and male patients. Additionally, we investigated factors affecting length of ICU and hospital stay in female patients. COPD, prolonged cross-clamping time, prolonged inotropic support, prolonged ventilator support, hemodialysis, postoperative blood transfusion (≥3 units), and postoperative stroke were the independent predictive factors in multivariate regression analysis of length of ICU stay in female patients.

Diabetes, previous stroke, prolonged cardiopulmonary bypass time, prolonged cross-clamping time, prolonged inotropic support, prolonged ventilator support, postoperative atrial fibrillation, postoperative renal insufficiency, hemodialysis, postoperative blood transfusion (≥3 units), and postoperative stroke were the independent predictive factors in multivariate regression analysis of length of hospital stay in female patients.

Several authors^13,21-24^ have concluded that female patients had equally or better late survival then compared with male patients. Abramov et al. stated that 5-year survival was longer in female patients than male patients (93.1% versus 90%).^9^ In our study, mid-term survival rate revealed no significant differences between female and male patients (93.9% versus 95.4%). The LITA usage were the independent predictive factors of survival in female patients. Calcification of ascending aorta, LITA usage, operation time, prolonged inotropic support, IABP usage were the independent predictive factors of survival in male patients. We hope the present study helps to more accurately describe postoperative and mid-term results of a surgical revascularization strategy, especially in an era where there may be other evolving treatment options for women, and also hind at how we might improve the differences observed in CABG results.

Conclusions. Advanced age, hypertension, diabetes, and hyperlipidemia were the independent predictive risk factors among female patients. Postoperative mortality was higher in female patients. Length of intensive care unit and hospital stay, and mid-term survival was similar between female and male patients.

## Acknowledgements

Authors have no a personal conflicts of interest. We do not have any financial relationship with a biotechnology, manufacturer, a pharmaceutical company, or other commercial entitly that has an interest in the subject matter or materials discussed in the manuscript.

## Ethical issues

This study was approved by our local Ethics Committee.

## Competing interests

Authors declare no conflict of interest in this study.
